# PFAS Environmental Pollution and Antioxidant Responses: An Overview of the Impact on Human Field

**DOI:** 10.3390/ijerph17218020

**Published:** 2020-10-30

**Authors:** Marco Bonato, Francesca Corrà, Marta Bellio, Laura Guidolin, Laura Tallandini, Paola Irato, Gianfranco Santovito

**Affiliations:** Department of Biology, University of Padova, 35131 Padova, Italy; marco.bonato@unipd.it (M.B.); francesca.corra@unipd.it (F.C.); marta.bellio@unipd.it (M.B.); laura.guidolin@unipd.it (L.G.); laura.tallandini@unipd.it (L.T.)

**Keywords:** antioxidant defenses, human health, environmental pollution, oxidative stress, epidemiology, perfluorinated substances, reactive oxygen species, toxicology

## Abstract

Due to their unique properties, perfluorinated substances (PFAS) are widely used in multiple industrial and commercial applications, but they are toxic for animals, humans included. This review presents some available data on the PFAS environmental distribution in the world, and in particular in Europe and in the Veneto region of Italy, where it has become a serious problem for human health. The consumption of contaminated food and drinking water is considered one of the major source of exposure for humans. Worldwide epidemiological studies report the negative effects that PFAS have on human health, due to environmental pollution, including infertility, steroid hormone perturbation, thyroid, liver and kidney disorders, and metabolic disfunctions. In vitro and in vivo researches correlated PFAS exposure to oxidative stress effects (in mammals as well as in other vertebrates of human interest), produced by a PFAS-induced increase of reactive oxygen species formation. The cellular antioxidant defense system is activated by PFAS, but it is only partially able to avoid the oxidative damage to biomolecules.

## 1. Introduction

Oxygen (O_2_) is an essential molecule for the life of many organisms present on Earth as it participates in all the oxidation reactions that characterize aerobic cell metabolism, being the final acceptor in the electron transport chain that leads to the formation of ATP at the mitochondrial level. During these processes, O_2_ is reduced to water, but some electrons can react directly with the molecule, forming intermediates called reactive oxygen species (ROS) [1].

It has been calculated that about 3% of the O_2_ used by the cell is improperly converted into ROS [2] but this rate of formation can easily increase in numerous physiological and pathological conditions such as physical activity, aging, cardiovascular disease, and cancer. Other factors favoring the formation of ROS are mechanical and thermal stress, exposure to ionizing radiation, hypoxia, and high partial pressure of O_2_, biotransformation of xenobiotic compounds, such as toxins, drugs, and chemical contaminants of various kinds [3,4,5,6].

Among contaminants of emerging concerns a particular interest is currently focused on Persistent Mobile Organic Contaminants (PMOC), which are highly polar compounds and likely to move and persist in the water cycle and in raw waters used for drinking water production and irrigation. Since they degrade very slowly, PMOC are extremely mobile in aqueous medium and their preferable accumulation matrix is water (including water contained in the biological tissues). For this reason, the exposure to PMOC can lead to serious health effects, which in many cases cannot be adequately assessed because the lack of monitoring and eco-toxicological data [7]. This is the case of the most important family of PMOC, which are the perfluorinated substances (PFAS).

The cytotoxic effects of ROS include peroxidation of membrane lipids with increasing malondialdehyde levels through oxidation of polyunsaturated fatty acids, alteration of redox balance, enzyme inactivation, and DNA damage [8,9]. All living organisms respond to a high presence of ROS by increasing the expression of antioxidant enzymes and many other molecules that are elements of the defense and repair toward oxidative damage [10]. Enzymatic antioxidants include proteins such as superoxide dismutase (SOD) [11,12], catalase (CAT) [13], glutathione peroxidase (GPx) [14], peroxiredoxins (Prdx) [15,16], and methionine sulfoxide reductases (Msr) [17]. These enzymes represent a first line against ROS, but do not guarantee complete coverage from the risk of oxidative stress because some compounds, generated by their reactions, are still reactive and potentially cytotoxic, such as the hydrogen peroxide (H_2_O_2_) produced by SOD. A joint and coordinated action of the various enzymatic components is therefore necessary to prevent further intracellular damage. The class of non-enzymatic antioxidants is represented by low molecular weight molecules rich in thiol groups such as metallothioneins [18,19] and glutathione (GSH), which are biosynthesized by the cell. The latter, in addition to being a substrate for GPx, can act directly as a scavenger of ROS during the detoxification processes of H_2_O_2_ and lipid hydroperoxides. There are also other compounds that are usually acquired through the diet such as ubiquinol, provitamin A, vitamins C, E, B6, B9, B12 and P, selenium, β-carotenoids, and polyphenols [20]. These molecules are mostly considered chain-breaking antioxidants since they interrupt the autocatalytic action of radical reactions, blocking the propagation of oxidative damage.

PFAS are a group of more than 4600 man-made chemicals with little or no data available on their biological effects [21]. The most studied and well-known PFAS are perfluorooctanoic acid (PFOA), perfluorooctane sulfonic acid (PFOS) perfluorohexanesulfonic acid (PFHxS) and Perfluorononanoic acid (PFNA) [22]. PFAS have unique physicochemical properties, due to their dielectrical properties, resistance to heat and chemical agents, low surface energy and low friction properties, and to their high stability thanks to the carbon-fluorine bond. These properties give to the substance versatility, strength, resilience, and durability, and that is why their use is convenient in a multiplicity of technical applications [23]. It has to be emphasized that to this date there is no evidence of biodegradation of perfluorinated carboxylic acids (PFCAs) and perfluorinated alkane sulfonates (PFAS) [24,25,26,27] that, in addition to their direct production, can also be the result of the terminal biodegradation products of perfluorooctanesulfonyl fluoride-based and fluorotelomer-based products [28]. In fact, the extensive uses of PFAS, together with their persistence, resulted in a worldwide dispersion, including in the biosphere, with a global distribution in living organisms like fish, birds, mammals, and humans [29].

This review summarizes the available data on the presence of PFAS in the environment and the effects they have on human health, with particular reference to the condition of oxidative stress. For this purpose, we have included the results of epidemiological studies, and those of in vitro and in vivo research correlating PFAS exposure and oxidative stress effects (in mammals as well as in other vertebrates of human interest).

## 2. PFAS and Their Distribution in the Environment

PFAS are a chemical family of compounds that have been produced since late 1940s [30]. They are found nowadays worldwide; they have been detected in oceans, across continents and in remote parts of the globe [31] including the North and South Pole [32,33,34,35,36,37,38], with several ecosystems affected in the USA, China, Africa, and Europe [39,40,41,42,43,44,45,46,47]. Their presence has been recorded in several different matrices, from the aquatic system to flora and fauna [48]. They are used in multiple industrial and commercial applications such as water repellent on clothing, leather, cosmetics, cookware, and paper, as well as being used as surface tension lowering agents in firefighting foam [49,50], due to their unique properties provided by the extreme strength of C–F bonds and their surfactant nature [51]. Recent studies have shown that some PFAS are toxic for both animals and humans [52]. Among the most common PFAS found in the environment (Table 1), PFOA and PFOS are considered some of the most widespread organic pollutants for biota and humans [44,53,54,55,56,57]. In addition, they are highly mobile once introduced to the aquatic environment [58] and are not removed by conventional wastewater treatment [59,60].

**Figure 1:**
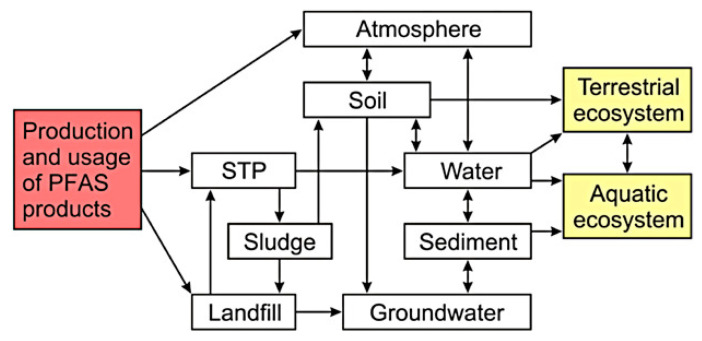
Pathways of PFAS into the environment and their fate. Adapted from Ahrens [53]. STP = sewage treatment plant.

Contamination of agricultural soil with PFAS can occur as a result of direct soil contamination, e.g., by fluorochemical industrial plants [82] or aqueous film–forming foams [83] through the use of biosolids [84] or contaminated irrigation water [31,85]. Consumption of contaminated food and drinking water is regarded as the main pathway of human exposure [86,87], especially the consumption of fish, meat, and eggs [88]. Several studies have examined the risks associated with PFAS exposure through contaminated food and water [89]. Drinking water may also be a dominant exposure pathway, if the water source is situated in a PFAS–contaminated area [90], but other possible pathways are summarized in Figure 1.

### 2.1. First Case: The USA

PFOA is found at low levels in the serum of most people living in the United States, with higher levels observed in occupationally exposed workers [16,91]. PFOA was reported to induce liver, testes, and pancreatic tumors in male rats over a 2-year period [92]. Because of PFOA long half-life, potential environmental persistence, and possible toxicity in human, there is a rising concern about whether it might be associated with human cancers [93]. Most previous human studies on the association between PFOA and cancer concerned mortality studies of occupationally exposed workers with few cancer deaths. One study has followed workers employed at a Minnesota PFOA production plant between 1947 and 1997 [94] and the results show that there are positive trends for prostate and pancreatic cancer across job categories with increasing PFOA exposure, but estimates were based only on 16 and 13 deaths, respectively [95]. A second mortality study has followed workers who had been employed at any time between 1948 and 2002 at the West Virginia DuPont Washington Works plant [96]. Steenland and Woskie [97] have recently updated this study and reported a significant increase in kidney cancer mortality with increasing estimated cumulative PFOA serum concentrations based on 12 kidney cancer deaths. We must remember that the DuPont chemical plant in Washington, West Virginia, began using PFOA in its manufacturing process in 1951. The plant began to release PFOA into the Ohio River and in the air in the 1950s, peaking in the 1990s, and decreasing emissions after 2001. PFOA emitted from the plant entered the groundwater, which was the public drinking water source [95]. Moreover, according to a 2004 study by ChemRisk Inc. (San Francisco, CA, USA), an industry risk assessor, between 1951 and 2003, the Dupont plant in Washington Works, West Virginia released more than 1.7 million pounds of C8 into the environment. This has led to an increase of the average PFOA blood concentrations of 32.9 ng/mL in the population inhabiting the six counties near the Dupont Washington Works plant [98] compared to the average PFOA blood concentrations found by the National Health and Nutrition Examination Survey 1999–2000 of 5.2 ng mL^−1^ [91]. Based on all these data, Barry et al. [95] conclude that PFOA exposure is positively associated with kidney and testicular cancer in this Mid-Ohio Valley population.

### 2.2. European Situation

Perfluorinated compounds (PFCs) are used to manufacture coatings for cookware and clothing, stain resistant carpets, food packaging, firefighting foams, paints, and adhesives, with additional uses in the photo, electronics, and aerospace industries [99]. Moreover PFAS-contaminated groundwater has been inadvertently used as drinking water supply in Sweden, Germany, the United Kingdom and Italy [100,101,102,103]. Within continental waters, large lakes present special features because of their physical characteristics, especially a long residence time, and the services they can provide to human populations. In fact, they are the main source of drinking water for residential populations [104]. Concerns about PFCs, particularly PFOS and PFOA are growing because they are globally distributed, environmentally persistent, bioaccumulative, and potentially harmful [105,106]. Up until 2002, approximately 4500 t of PFOS-related chemicals were produced worldwide annually, with a total historical production of about 100,000 tons [35]. From 2002 some users moved to alternative fluorine-based products, i.e., perfluorobutane sulfonate (PFBS) has been introduced as an alternative for PFOS [107]. This compound has recently been listed as chemical for regulation within the Stockholm Convention [108] and was banned in the European Union in 2007 for most applications [109]. In 2009, it was added as Annex B to the Stockholm Convention on Persistent Organic Pollutants (POPs). In 2015, more than 200 scientists signed the Madrid Statement [110], requiring the international community to cooperate in limiting the production and use of PFAS, and raising concern about the new short-chain perfluorinated compounds. PFOA was listed on Annex A of the Stockholm Convention in 2019 [111] and PFHxS has been proposed for listing under the Stockholm Convention and is currently under review [112]. In Europe, as well as in USA, a single European drinking water regulation for both PFOA and PFOS has not been established yet, and each member of European Union has a different regulation. The UK Health Protection Agency (HPA) set the PFAS levels in drinking water at 0.3 mg·L^−1^ for PFOS and 10 mg·L^−1^ for PFOA [113]. Other Regulatory Institutes set limits in terms of tolerable daily intake (TDI). The European Food Safety Authority (EFSA) indicated 0.15 mg·kg^−1^ body weight (bw) for PFOS and 1.5 mg·kg^−1^ bw for PFOA as the limit [114]. The German Commission for Drinking Water [115] set the limit to 0.1 mg·kg^−1^ bw for both PFOS and PFOA. In Italy, on January 2014, the Italian National Health Institute (ISS) indicated the following performance limit: PFOS ≤ 30 ng·L^−1^; PFOA ≤ 500 ng·L^−1^; and other PFAS ≤ 500 ng·L^−1^.

### 2.3. Veneto Region

**Figure 2:**
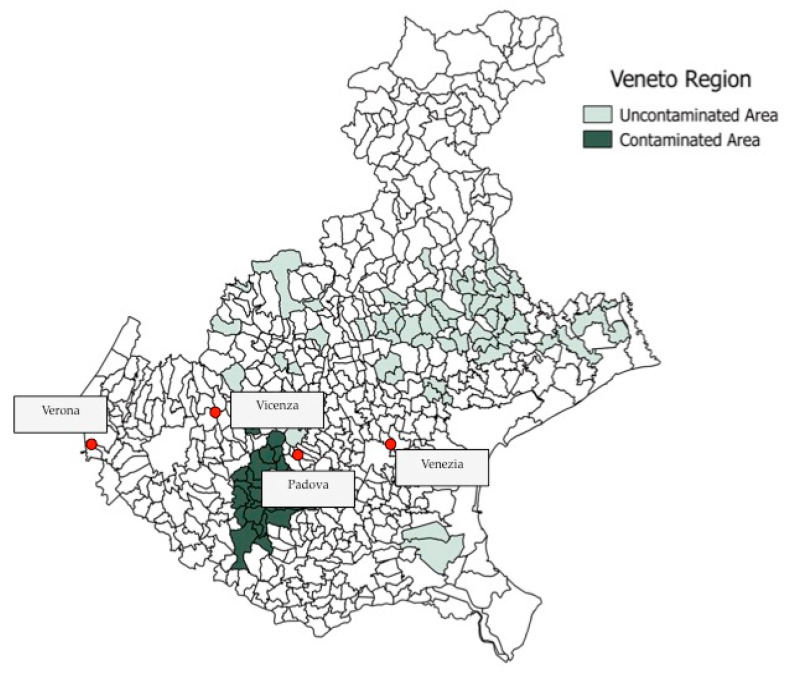
Contaminated (in dark grey) and uncontaminated (in light grey) municipalities on the basis of the Italian National Health Institute (ISS) performance limits in drinking water in the Veneto region. In many municipalities (in white) no analyzes were carried out [120].

The Po valley has a strong agricultural history and is responsible for the success of the Veneto region as one of the most significant producers of fruits, vegetables, cereals and wine in Italy and Europe [116], making it among the most important economic sectors in the region. In general, Italy has the highest use of water for agricultural purposes in Europe, about 50% of which is in the form of surface and groundwater [117]. In 2013, a large-scale contamination of PFAAs was discovered in the Veneto region, Northern Italy, as a consequence of the emissions of a fluorochemical plant in the province of Vicenza [118] showed in Figure 2. The municipalities considered as “contaminated areas” were selected from the Veneto Region report of 2015, which listed municipalities with PFOS, PFOA, and the other PFAS exceeding at least one of the ISS performance limits of 30 ng·L^−1^ for PFOS, 500 ng·L^−1^ for PFOA and 500 ng·L^−1^ for the other PFAS in drinking water but did not report PFAS concentration level [103]. The white areas were not selected in the Veneto Region Report.

In the Vicenza province, concentrations of PFOA up to 20 µg·L^−1^ were detected in the groundwater, 3.4 µg·L^−1^ in surface waters and 7.9 µg·L^−1^ in spring waters of the Vicenza province were detected, with measured peaks up to 700 µg·L^−1^ in industrially contaminated wells in the area [119]. According to Pitter et al. [103] residents of a vast territory of the Veneto Region have been inadvertently exposed to drinking water containing high concentrations of PFAS. Through public waterworks, contaminated groundwater was provided to roughly 140,000 people. This contamination is one the largest cases of high residential exposure to PFAS ever reported, and it resembles in origin, extent, and characteristics the one that occurred in the Mid-Ohio Valley, in the United States [98]. PFOA reached the highest concentrations both in drinking water and serum, consistent with previous reports from the Mid-Ohio Valley [98]. A recent study has showed that communities living near PFOA chemical plant have higher risk of diabetes, cerebrovascular diseases, myocardial infarction and Alzheimer’s disease in both sexes and for kidney cancer, breast cancer, and Parkinson’s disease in females [120]. Moreover, the effects of PFAS as endocrine disruptors are manly connected with hormonal unbalances, fertility decrease, birth outcomes defects [121], and alterations of many biochemical parameters, not inducing fatal outcomes [122].

## 3. Epidemiological Studies

In order to describe the interactions with the human population, a large number of biomonitoring, basic mechanistic and epidemiological studies have been increasingly developed worldwide since the early 2000s when the PFAS were found to be environmentally ubiquitous [10,123] but especially, during the last 10 years [124]. Before, the pioneristic works accomplished by Taves in the 1960s and in the early 1970s [125,126,127,128] about the presence of PFAS in human tissues, had little resonance. Studies on the PFAS presence and health status in people working in PFCs plants were also performed. Ubel et al., found higher than normal levels of organic fluorine in the blood of workers exposed to fluorochemicals in an industrial environment without ill health effects attributable to exposure to fluorochemicals [129]. The analysis of clinical examination data from PFOA production workers found clear associations between increased PFAS concentrations in the blood and decreased leukocyte counts, suggesting that cell-mediated immunity could be affected by PFOA [130,131]. In a study conducted on the mortality rates of workers exposed to PFOA no associations were seen in male employees between any cause of death and length of employment, but a standardized mortality ratio of 2.03 for prostate cancer was seen in exposed males compared to 0.58 in non-exposed males. A weak but significant positive association was seen between exposure and prostate cancer risk and the risk increased with the length of employment. A positive association was seen as well between age at first employment and prostate cancer mortality [132]. In a subsequent study, 115 occupationally exposed workers were examined for the cross-sectional associations between PFOA and hepatic enzymes, lipoproteins, and cholesterol. The findings indicated that no significant clinical hepatic toxicity was observed in the study at those PFOA levels, leading to the possibility that PFOA may modulate the previously described hepatic responses to obesity and xenobiotics [133]. Additionally, in 1976, 3M began testing for—and finding—PFOA in workers’ blood (men and workers pregnant women) always with internal reports. Only in 1999, 31 years after Taves’ initial discovery, 3M communicated with a Report to U.S. EPA the data about PFAS accumulation in humans [134]. Thus, it appeared also that from the 1950s until the early 2000s, the chemical plant, DuPont Washington Works, in southwest Parkersburg, WV, USA, released PFOA into the air and in the Ohio River. At that time it was named “C8” as PFOA, perfluorooctanoic acid, chemical formula C_8_HF_15_O_2_, is constituted by a chain of eight carbons, seven of them saturated with 15 fluorines, and the eighth in form of carboxylic acid (Table 1). Thanks to the emissions, PFOA reached drinking-water supplies by entering the groundwater where it was detected in six districts near the DuPont plant in 2002. This event gave rise to the famous class-action lawsuit done against Dupont by the residential population. Approved in February 2005 by West Virginia Circuit Court the class action obtained, as well as the carrying out of a water supply clean-up, the conduction of a biomonitoring study (the C8 Health project) and the establishment of the C8 scientific panel for the subsequent epidemiological health evaluations.

### 3.1. The C8 Health Project

As introduced above, the C8 health project, conducted between August 2005 and July 2006, collected interviews and blood samples, from about 69,000 people living near the 3M plant through a biomonitoring study. The mean levels for serum PFOA resulted 500% higher than previously reported for a representative American population [91,98,135]. On the basis of the biomonitoring survey and the scientific data available at that time, the C8 scientific panel developed an important series of exposure and health epidemiological studies during 2005–2013, in the Mid-Ohio Valley communities, potentially affected by the releases of PFOA (or C8) emitted since the 1950s from the Washington Works plant in Parkersburg, West Virginia. Six probable cause-effect links were found between C8 and pregnancy-induced hypertension/preeclampsia, autoimmune ulcerative colitis, thyroid disease, teste and kidney cancers, and increased plasma cholesterol levels. The C8 panel study concluded that there was an absence of a probable link between exposure to C8 (PFOA) and chronic kidney disease, liver disease, osteoarthritis, Parkinson’s disease, autoimmune diseases other than ulcerative colitis (i.e., rheumatoid arthritis, lupus, type1 diabetes, Crohn’s disease, or multiple sclerosis), common infections, including influenza, in children or adults, neurodevelopmental disorders in children, including attention deficit disorders and learning disabilities, asthma or chronic obstructive airways disease, stroke, and Type II diabetes. [136]. The C8 Science Panel has completed its work in 2013, and no longer exists. Oxidative stress problems were not studied in the C8 panel activities. The discussion on PFAS risks, strongly reinforced after the Ohio accident, the results of C8 health project and the links between PFOA and human health detected by the C8 scientific panel, gave rise to three important statements:

- Helsingør Statement on Poly- and Perfluorinated Alkyl substances (PFAS) [137];
- The Madrid Statement on Poly- and Perfluoroalkyl Substances (PFAS) [110];
- Zürich Statement on Future Actions on Per- and Polyfluoroalkyl Substances (PFAS) [138].
- These statements increased the consciousness of researchers, managers and communities on the PFAS issues.

Plenty of studies were developed in the following years both by the C8 Panel scientists with collaborators (http://www.c8sciencepanel.org/publications.html, for a total of 29 additional papers up to Jan 2020) and by a large number of researchers worldwide, looking to enhance the knowledge about the risk of PFAS on human health.

### 3.2. Studies after the C8 Health Project and Panel

In the following years the cause-effect links highlighted by the C8 panel were confirmed and other correlations were also studied. However, due to the intrinsic difficulties in developing epidemiological significant data on human pathologies, a major part of the studies was focused on the PFAS concentrations in cohorts of residential exposed populations. Limited information have been collected on PFAS accumulation in solid human tissues (e.g., adipose and liver). The major part of the studies reported data on PFAS levels in blood serum and breast milk and were mostly dedicated at the presence and concentrations of PFOA and PFOS, but other studies included also PFBS, PFHxS, PFDS, PFBA, PFPeA, PFHxA, PFHpA, PFNA, and PFDA.

In the U.S. general population, PFAS were detected in more than 99% of all serum samples from the 2009–2010 National Health and Nutrition Examination Survey (NHANES) [139]. The concentrations of PFOS and PFOA, the two most widely detected PFAS, showed a decrease in many countries following the drop in production after the year 2000 [140] but exposure to other short-chain and also other long-chain PFAS are increasing in U.S. as in many countries.

Investigation on plasma samples, collected in Sweden in the years between 1987 and 2007, showed PFOS and PFOA blood concentrations peaking around 2000 and then declining, while increasing PFHxS, PFNA, PFDA, and PFUnDA concentrations within the overall study period [141]. This tendency was confirmed by reported increasing concentrations of PFBS, PFHxS, PFNA, and PFDA in Swedish blood serum samples in a cohort of 413 primiparous women between 1996 and 2010 [142]. This is also in line with the study from Gebbink et al. [143] reporting increasing trends in pooled serum samples from Sweden for PFHxS, PFNA, PFDA, PFUnDA, PFODA, and PFTrDA and with analyses of serum samples from Norway from 1979 to 2007 that documented decreasing concentrations of PFOS and PFOA from 2001 onwards, whereas PFNA, PFDA, and PFUnDA were increasing, while for PFHxS and PFHpS no trend could be observed [144].

In Denmark, the concentrations of seven PFAS (PFHXS, PFHpS, PFOS, PFOA, PFNA, PFDA, and PFUnDA) decreased in the period 2008–2013 [145]. Schröter-Kermani et al. [146] reported decreasing concentrations from 2001 onwards for PFOS, from 2008 for PFOA and from 2005 for PFHxS and stable concentrations for PFNA in samples from Germany from 1982 to 2010. Also, Yeung et al. [147,148] observed decreasing concentrations for PFOA after 2000, but increasing concentrations for PFNA, PFDA, and PFUnDA. Decline in perfluorooctane sulfonate and perfluorooctanoate serum concentrations were reported in an Australian population from 2002 to 2011 [149]. In North Rhine-Westphalia, Germany, where 40,000 inhabitants were exposed to PFAS-contaminated soil conditioners used on fields, a cross-sectional study assessed the levels of PFAS contamination in 170 children (5–6 years of age), 317 mothers (23–49 years), and 204 men (18–69 years) and found that PFAS concentrations in blood plasma of children and adults exposed to contaminated drinking water were increased 4-to 8-fold compared with controls [150]. In Japan and Korea, PFOS was detected in human serum samples collected from 1994, 2000, 2003–2004, and 2007–2008 [151,152] with significant higher levels (*p* < 0.01) in samples from the urban region than the rural region, and with gender related discrepancies for significantly higher (*p* < 0.05) concentrations of PFOS in males than females. Higher PFOS concentrations were similarly observed in urban and rural zones in China, with significantly higher concentrations in males than females [153,154].

In Italy, a study by Ingelido et al. [155] looked at PFAS biomonitoring data on blood samples to characterize the extent of exposure to PFCs in groups of the Italian general population residing in two different urban location. Analyses were carried out on blood samples collected in 2008 from 230 subjects residing in Rome, in the Lazio region, Central Italy (182 subjects), and in Brescia, an industrial town located in the Lombardia region, Northern Italy (48 subjects). PFOS and PFOA were detected and quantified in all samples. PFOS concentrations ranged from 0.06 ng·g^−1^ to 29.6 ng·g^−1^, PFOA concentrations from 0.22 ng·g^−1^ to 51.9 ng·g^−1^. The medians, geometric means, and arithmetic means were 6.31, 5.77, 6.86 ng·g^−1^ for PFOS and 3.59, 3.32, and 4.15 ng·g^−1^ for PFOA, respectively. The whole age range was 20–65 years, shared in three subgroups, 26–35, 36–50, and 51–65. Overall, PFOS concentrations were consistently higher than those of PFOA in all age groups. Concentrations of PFOA and PFOS were higher in males than in females across all age groups. This agrees with studies carried out in the USA [91,156], Australia [157], China [153], Japan [158], and Germany [159,160]. The strong correlation observed between PFOS and PFOA concentrations is in line with observations reported in other studies [135,160,161,162]. The observed correlation could be partly due to the uptake of both compounds from common sources or pathways (food and migration from food packaging, drinking water, indoor dust) [157,163,164,165]. Age-dependence of PFC concentrations is plausible to be related to the persistence and potential for bioaccumulation [29] of these chemicals, associated with conditions of constant exposure. The concentrations measured in the study, resulted to be among the lowest observed worldwide, and they were comparable with those observed in a group of subjects (50 adult donors, 42 males, and 8 females) enrolled in the town of Siena, Tuscany, Central Italy, in 2001 [162], and were confirmed by a later work realized within the WomenBioPOP project in six Italian regions [166].

One-third of households in Ronneby, Sweden (28,000 inhabitants), were supplied with drinking water contaminated with PFAS from firefighting foams used in a nearby airfield [167]. After the elimination, in 2013, of the contaminated drinking water supply, for two years (2014–2016) a total of 3418 people from Ronneby participated in the study and a reference group of 242 subjects from a nearby unexposed municipality (Karlshamn) was also examined in 2016. In that way was possible to evaluate the half live of different PFAS, the median value of the remaining half-lives for PFHxS was 5.5 years (5–95% range: 3.0–9.2 years). For PFOS, the median half–life was 3.5 years (5–95% range: 2.2–6.2 years). The median half–life for PFOA was 2.7 years (5–95% range: 1.8–5.1 years). Serial analyses of serum samples from former 3M production workers after retirement suggested elimination half-lives for long-chain PFAS to be three years (PFOA) and five years (PFOS). Declines in serum-PFOA concentrations after elimination of the water contamination suggest a median elimination half-life of 2.3 years, thus confirming the persistence of PFAS in the human body.

The case of the impressive Water Contamination by PFAS in the Veneto region, [118,168,169], principally ascribed to the Rimar-Miteni (RM) plant in Trissino-Vicenza led the Region Administration to design an ongoing health surveillance plan for people resident in the PFAS Impacted Area (21 + 9 Municipalities) in order to aid in the prevention, to get early diagnoses and treatment for some of the chronic disorders with epidemiological evidence of association with PFAS exposures, i.e., dyslipidemia, hyper-tension, diabetes mellitus, liver dysfunction, metabolic syndrome, kidney dysfunction, and thyroid disorders. The case requires a series of epidemiological studies on the distribution and effects of PFAS.

Ingelido et al. [170], in a study dedicated to the Veneto region areas under PFAS impact, involved 507 subjects. Of these, 257 were “Exposed” (“E”), residing in Veneto Region municipalities at high PFAS exposure, and 250 “Not Exposed” (“NE”), residing in municipalities in areas at presumed PFAS background exposure. PFAS serum concentration median value of the “E” group (13.77 ng g^−1^) was 8 times higher than the median value of the “NE” group (1.64 ng·g^−1^). PFOA was the analyte that presented the larger data dispersion in the “E” subjects with the P95 value that resulted 18 times higher than the median. Males showed PFAS serum concentrations more than 3 times higher (26.07 ng·g^−1^) than females (7.88 ng·g^−1^), both in the “E” and the “NE” group. Differently from sex, age did not seem to strongly affect the levels of PFAS, confirming other previously reported data [171,172,173,174]. A following study in the Veneto region has been conducted on a cohort of 18,345 participants (49.1% females and 50.9% males) born between 1978 and 2002, i.e., 14–39 years of age at the recruitment time in the municipalities impacted by the PFAS contamination [103]. The PFAS with the highest serum concentrations were PFOA. The median serum PFOA concentration in the study (44.4 ng·mL^−1^) was substantially higher in comparison to the concertation found in the C8 Health Project (28.2 ng·mL^−1^) [98], and 27 times higher than the median serum level (1.64 ng·mL^−1^) of not exposed residents of the Veneto region [170]. The major predictors of serum levels were gender, municipality, duration of residence in the affected area and number of deliveries. Interindividual variation of serum PFAS levels was partially explained by the considered predictors.

The long PFAS half-lives and the duration of exposure time in the exposed populations are the baseline of epidemiological studies to evaluate long-term health and mortality effects in workers and residential cohorts associated with exposure to perfluoroalkyl and polyfluoroalkyl substances. In a cohort of 5791 workers at the DuPont chemical plant in West Virginia, Steenland, and Woskie [97] found evidence of positive exposure-response trends for malignant and nonmalignant renal diseases. These results were limited by low sample size and restriction to mortality data.

Girardi and Merler [175], comparing two cohorts of workers, the first one in a PFAS production plant (the RM plant in Trissino-Vicenza), the second one devoted to the railroad material maintenance, found a clear excess of overall mortality and of mortality for liver cancer, liver cirrhosis, malignant neoplasm of lymphatic and hematopoietic tissue among the subjects working at PFAS plant or in the highest cumulative PFOA concentration category. Their findings suggest an association between PFOA exposure and the risk of mortality not previously reported. Induced tumors have been ascribed to non-genotoxic mechanisms, such as an indirect genotoxic hazard, such as the ability of PFOA to induce oxidative stress, even if recently denied in an assessment on mice [176]. However there are no doubts of toxic effects on liver experienced by the workers under study: The surveillance of the RM factory workers showed a positive association for alanine aminotransferase (ALT) and γ-glutamyl transferase (GGT) hepatic enzymes and an inverse association with bilirubin [177]. A longitudinal study [178] on a cohort of 1002 individuals from Sweden (50% women) at ages 70, 75, and 80 in 2001–2014, confirmed these observations, showing that exposure and biological persistence to PFAS caused liver dysfunction and toxicity, measured in terms of changes in ALT and alkaline phosphatase (ALP) levels. Furthermore, PFAS with long perfluoroalkyl chains are associated with increased GGT and decreased bilirubin levels, an important antioxidant and cytoprotectant, decreased levels of which have been associated with adverse health effects, including liver dysfunction, cardiovascular disease, and the metabolic syndrome.

At the present moment, relatively few studies have investigated the associations between plasma PFAS concentrations and markers of liver function. Most of them found evidence of a relationship between PFAS concentrations and liver enzymes including ALT, ALP, and GGT. Increased ALT activity is clinically used as a marker of liver dysfunction. Concentrations of PFHpA, PFOA, PFNA, and PFOS were positively associated with the activity of ALT in a large cross-sectional study on 47,092 Mid-Ohio Valley residents with elevated PFOA concentrations due to a drinking water contamination [179]. A positive association between PFOA and ALT was also reported in an ancillary study on 30,723 Mid-Ohio Valley residents [180]. In all people falling into the third tertile of cumulative internal dose of PFOA (which exceed the lowest-observed-adverse-effect level, estimated to cause liver toxicity in experimental animals [181]) liver toxicity was actually observed [180]. The IARC Monograph on PFOA noted that liver toxicity observed in rodents has been associated with both PPARα–dependent and independent mechanisms. The in vitro data from human are consistent with tests on multiple molecular pathways [182]. Hepatic effects of PFOS are clearly PPARα-independent [176].

Looking at previous studies in animal and cell models, in which the increased oxidative stress resulted as an important response to PFC exposure [183,184,185,186], the influence of low-level environmental PFC exposure on serum metabolome was studied in a cohort of 181 adult males individuals (males, in order to avoid the periodical female endocrine changes) from the general population was studied [187]. In particular, serum samples were investigated for different PFCs concentrations and metabolome changes. PFCs species relations to ten serum biomarkers were screened based on their internal doses. Eight biomarkers (i.e., D-glucurono-6,3-lactone (DG63L), α-carboxyethyl hydroxychromanol, arachidonic acid, hypoxanthine, oxoglutaric acid, pyroglutamic acid, tetrahydrobiopterin, and xanthine) were negatively associated with the high exposure levels and two biomarkers (i.e., deoxyarabinohexonic acid and hydroxybutyric acid) were positively associated. PFC exposure resulted associated with the disturbance in GSH cycle, TCA cycle, nitric oxide (NO) generation, and purine oxidations. In fact, the obtained serum metabolic biomarkers implied that the lipid metabolism, xenobiotic detoxifying, anti-oxidation, and NO signal pathways may be affected by PFC exposure in a dose dependent trends between serum pollutant residuals and metabolic biomarkers. Oxidative stress may result in a distal linkage of DG63L to ascorbate. The observed alteration of oxoglutaric acid may further support the response of DG63L to PFC-induced fatty acid oxidation via ascorbate and aldarate metabolism pathway.

## 4. Mammals: In Vitro and In Vivo Studies Correlating PFAS Levels and Oxidative Stress

A certain amount of oxidative damage takes place even under normal conditions, such as aging. Cells act to counteract the oxidant effects and to restore redox balance. Cells react by resetting critical homeostatic parameters are reset, activating or silencing genes encoding defensive enzymes, transcription factors, and structural proteins. A consequence of this action can be the accumulation of oxidation products of lipids, nucleic acids, proteins, sugars, and sterols causing cellular dysfunction [188]. Oxidative stress occurs when oxidative action of ROS is stronger than antioxidant responses of the cellular defense system, and it usually determines peroxidation of membrane lipids, DNA damage and mutations, oxidation and deactivation of proteins and enzymes, and activation of apoptotic and/or necrotic processes.

ROS production is a common denominator in many pathological conditions and can lead to severe cellular damage leading to physiological dysfunction and cell death. Oxidative stress has been observed in relation to several diseases in humans, including diabetes, atherosclerosis, cardiovascular diseases, chronic inflammatory diseases, central nervous system disorders, age related disorders, and cancer [189,190,191,192], and has been linked to a shortening of life [193].

Oxidative stress can be also induced by exposure to environmental pollutants such as dioxins and heavy metals [194,195], and a similar effect can be relevant for PFAS [196]. As mentioned above, exposure to PFOS and PFOA is known to cause an increase in ROS formation and, consequently, cellular oxidative stress (Table 2). Oxidative stress and physiological process disruption based on fatty acid similarity are widely studied mechanisms of PFAS toxicity [197].

### 4.1. PFAS Affect Oxidative Stress Biomarkers In Vitro

PFAS are suspected carcinogens and the fact that they generate oxidative stress can be a potential action mode. Hu and Hu [198] reported a study in which they analyzed single antioxidants enzyme activity after exposure to PFAS in hepatoma Hep G2 cells. Exposure to PFOA and PFOS resulted in an increased activity of SOD, CAT, and glutathione reductase (GR) and decreased activity of GPx and glutathione S-transferase (GST). The authors suggested that the observed changes in activities of antioxidative enzymes indicated that PFOA and PFOS may overwhelm the balance of the antioxidant system, boost the generation of ROS, impact the mitochondria, and result in the initiation of apoptosis program.

Wielsøe et al. [196] conducted a study in which the results indicate a possible genotoxic and cytotoxic potential of the PFAS in human liver cells inducting oxidative stress.

In particular, they investigated the effect on oxidative stress factors of seven long chained PFAS: PFHxS, PFOA, PFOS, PFNA, PFDA, PFUnA, and PFDoA in human hepatoma cell line (HepG2) and an exposure time of 24 h using concentrations from 20 nM to 200 μM (much higher than the environmental ones).

Four of the PFAS (PFHxS, PFOA, PFOS, and PFNA) showed dose-dependent increase in DNA damage determined by the comet assay. A high level of DNA lesions may lead therefore to mutagenesis, cytostatsis, and cytotoxicity [199].

Six PFAS (except for PFDoA) increased ROS generation and the increase was dose-dependent for two PFAS (PFHxS and PFUnA).

PFOA significantly decreased the total antioxidant capacity (TAC), whereas a non-significant trend in TAC decrease was observed for PFOS and PFDoA and an increase tendency for PFHxS, PFNA and PFUnA.

Among the tested PFAS, five were PFCA and two sulfonic acids (PFSA). The two investigated PFSA (PFHxS (C6) and PFOS (C8)) both increased the DNA damage dose dependently and induced intracellular ROS generation, but none of them affected the TAC level significantly. For the five PFCA (PFOA, PFNA, PFDA, PFUnA, and PFDoA) with carbons chains containing between 8 and 12 carbon atoms, the length of the carbon chain seemed be related to some degree to all the three endpoints with the highest potency of the shortest carbon length.

PFOA increases oxidative stress and mitochondrial dysfunction that lead to apoptosis and cytotoxicity in rat β-cell-derived RIN-m5F cells [200]. These mechanisms may contribute to the effects of subchronic, elevated exposures to PFAS on thyroid hormone homeostasis, liver toxicity, and body weight observed in animal experiments [29,181].

PFAS have been shown to induce adipocyte differentiation and lipid metabolism in cell culture [201]. Recent evidence from in vitro studies has further demonstrated potentially estrogenic and antiestrogenic effects of PFAS [202,203].

All these results indicate a potential genotoxicity and cytotoxicity of PFAS in human cells.

### 4.2. PFAS Affect Oxidative Stress Biomarkers In Vivo

PFAS are suspected obesogens, and the obesogenic potential of PFAS has been shown by in vitro and in vivo studies.

In vivo experiments in mice showed that PFOA induced histopathological changes in the pancreas through the increasing of oxidative stress [204].

PFAS have the ability to activate the peroxisome proliferator-activated receptor alpha (PPARα) and induce peroxisome proliferation in rodents, but the human relevance for this mode of action has been questioned [205,206].

Experimental studies and animal models have shown that PFAS can act as agonists of the PPARα, which might lead to liver damage [29,207,208]. In particular, these studies have found that PFAS activate PPARα [65,209] and PPARγ [209,210,211], which regulate energy homeostasis, lipid and glucose metabolism, and adipocyte differentiation and function [212].

PPARα plays a crucial role in lipid homeostasis and in the proliferation of peroxisomes, cell organelles involved in lipid metabolism and in the conversion of ROS in less harmful compounds (reduction of hydrogen peroxide). Upregulation occurs because PFOA (as a structural analogue of endogenous fatty acids) can be transported to the nucleus, where it activates PPARα. Consequently, peroxisomal proliferation is a process involving oxidative stress and is a specific biomarker of exposure to many xenobiotics.

## 5. Oxidative Stress in Human Health Due to PFAS Pollution

PFAS exposure has been associated with multiple adverse health effects in humans (Table 2), including perturbations in thyroid [213,214,215], kidney [216,217], and metabolic function [218,219,220]. Because, as already mentioned, PFAS are known to persist in the body and in the environment for years, biomonitoring efforts focused on highly exposed populations with long-term periods of follow up would provide invaluable information on the health risks imposed by this chemical class.

In adults PFAS interfere also with reproductive [221] and steroid hormones [222], adipokines [223], asthma and allergies [224], and maternal fatty acids [225].

In general, in adult people lung tissue is thought to accumulate the highest concentration of PFAS, although PFOS and PFOA tend to accumulate predominantly in the liver and bone structure, respectively. PFOA, PFOS, PFNA, PFDA, and PFUnA were found in all human tissues [226].

### 5.1. Male Infertility

Endocrine disruptors (EDs) are exogenous substances able to impair endocrine system; consequently, they may cause numerous adverse effects. Over the last years, particular focus has been given to their harmful effects on reproductive system, but very little is known, especially in males. A study of Cargnelutti et al. [227], aimed to discuss the detrimental effects of EDs exposure on fetal testis development, male puberty and transition age, searching in the existing literature. According to WHO/IPCS 2002 definition, “an endocrine disruptor (ED) is an exogenous substance or mixture that alters functions of the endocrine system and consequently causes adverse effects in an intact organism, or its progeny”. Examples of these molecules are phthalates, bisphenol A (BPA), dichlorodiphenyldichloroethylene (DDE), polychlorinated biphenyls (PCBs), and, of course, per- and poly-fluoroalkyl substances (PFAS).

EDs mimic naturally occurring hormones like estrogens and androgens, and exert their toxicity by interfering with the normal hormonal homeostatic mechanisms (including pre- and postnatal development [228]) that promote growth and development of tissues, interfering with the hormonal binding to the corresponding receptor, notably the androgen receptor (AR) or the estrogen receptor (ER) [229]. Moreover, EDs exposition brings to an inflammatory response which triggers leukocytes and macrophages (present in the prostate and in seminal vesicles) to generate ROS about 100 times more than it is produced under normal conditions (just think about spermatozoa which possess numerous mitochondria in the mid-piece of the flagellum in order to produce the immense amount of energy needed for motility) [230]. Therefore, EDs may have negative effects not only on exposed individuals, but also on their offspring and on future generations.

The effects of EDs on the male reproductive system are usually attributed to the interactions of these chemicals with the normal production and/or function of steroid hormones that are responsible for the masculinization of the Wolffian ducts. Testis development during fetal life is crucial for male reproductive function in adulthood. Indeed, fetal period is critical for the regular development of the testis and is known as a period of high sensitivity to many EDs [227]. Both functions of testis (spermatogenesis and steroidogenesis) are set up early during fetal life, involving Sertoli cells development and Leydig cells differentiation. In particular, Leydig cells are responsible for testosterone production (responsible for masculinization) [227] which can be compromised by the presence of xenobiotics, as PFASs, and so altering ROS production.

In rats, PFOA does not seem to affect fetal Sertoli cells but may increase tendency of apoptosis in fetal Leydig cells. This damage seems to affect both proliferation and differentiation of stem Leydig cells or their progeny [227,231]. An altered redox environment is characteristic of the aging of many cell types, including Leydig cells, which steroidogenic function is regulated in part by luteinizing hormone (LH). Beattie et al. [231] study demonstrates how LH stimulation resulted in a significant increase of genes associated with stress response expression, antiapoptotic pathways and ROS production which brings to DNA damage (but also proteins and lipids). LH binds to G protein-coupled receptors, thereby initiating a cascade of events that include activation of adenylate cyclase, increased intracellular cAMP levels, activation of cAMP-dependent protein kinase, phosphorylation of proteins, and transfer of cholesterol to the inner mitochondrial membrane. Superoxide anions and other ROS are produced by the mitochondrial electron transport chain during the process of oxidative phosphorylation. Leydig cells, in addition, contain cytochrome P450 enzymes that catalyze the oxidation of metabolic intermediates in the steroidogenic pathway and in doing so can leak electrons and serve as a source of free radical generation. Cells contain enzymes and other molecules that can neutralize or scavenge ROS.

Regarding PFOS, it seems to damage Sertoli cells by perturbing actin cytoskeleton in primary cultures of rodent and human and may directly inhibit pubertal development of rat Leydig cells. In humans, prenatal PFOS exposure may increase fetal steroid hormone production, although no association with cryptorchidism or hypospadias has been observed [227].

Alterations of testosterone concentrations could also have detrimental effects on ROS generation in the vascular system. In a review written by Tostes et al. [232], testosterone has been shown to increase ROS in smooth muscle cells via different cellular sources, such as activation of NAD(P)H oxidase, mitochondria, cyclooxygenase 2 (COX–2), and xanthine oxidase. The genomic action of testosterone induces c-Src and PI3K/Akt pathways which, in turn, activates NAD(P)H oxidase and xanthine oxidase, respectively. Testosterone may also increase ROS, via its nongenomic action, through GPRC6A receptor. Increased ROS production may lead to migration, apoptosis, hypertrophy, and inflammation causing vascular dysfunction [232].

Darbandi et al. [230] observed that EDs, as PFCs, could induce excessive production of ROS beyond that of cellular antioxidant capacity, thus causing oxidative stress. In turn, oxidative stress negatively affects male reproductive functions and may induce infertility either directly or indirectly by affecting the hypothalamus–pituitary–gonadal axis and/or disrupting its crosstalk with other hormonal axes [233,234,235,236]. A direct effect could be the production of superoxide anions by the NADH-dependent oxidoreductase and NAD(P)H-oxidase present respectively in the inner membrane of the mitochondria (present in the midpiece of spermatozoa) and in the plasma membrane [237,238]. In the plasma membrane of germ cells, H_2_O_2_ was also found, an uncharged molecule which is membrane permeable and which can lead to the production of other ROS [238]. An excessive ROS production may lead to either apoptotic or necrotic cell death [239]. On the other hand, EDs can indirectly cause the enhance of the circulating cortisol level, leading to oxidative stress induction and reducing circulating testosterone levels. Increased cortisol decreases LH secretion through crosstalk between the hypothalamus–pituitary–gonadal and hypothalamus–pituitary–adrenal axes [230]. Decreased LH concentration fails to stimulate the Leydig cells resulting in decreased testosterone production, whereas decreased FSH affect normal Sertoli cell functions. These toxicants also interfere with the cellular communications and adhesions between Sertoli–Sertoli cells and Sertoli–germ cells via the PI3K/c–Src/focal adhesion kinase signaling pathway that leads to reproductive dysfunction and disrupted hormonal secretion [230].

There are controversial results about these studies, but PFAS are for sure endocrine disruptors that compromise many processes and alter redox environment.

### 5.2. Female Infertility

Polycystic ovarian syndrome (PCOS), one of the main reasons of the ovarian infertility, is a common endocrine disorder in reproductive age women. In a study of Wang et al. [240] blood concentrations of ten PFAS were measured and associated with 180 infertile PCOS-cases and 187 healthy controls recruited from the Center for Reproductive Medicine of Shandong University (China). The plasma concentration of the perfluorododecanoic acid (PFDoA), 12 carbons lengths of perfluorocarboxylic acids, was associated with a significantly increased risk of PCOS-related infertility. Of course, PCOS is accompanied with many disturbances in hormone synthesis and antioxidant defense. Masjedi et al. [241] investigated the effects of vitamin D on steroidogenesis, apoptosis, ROS production and antioxidant defenses on human normal granulosa cells (N–GCs) and granulosa cells from polycystic ovaries (PCO–GCs). In particular, basal ROS production in PCO–GCs was markedly greater than that of N–GCs (which was attenuated by vitamin D treatment) and consequently cell apoptosis was correlated with ROS production which could be enhanced by the presence of pollutants such as PFAS. Antioxidant defenses were instead compromised; indeed basal expression and activity of GPx were significantly lower in PCO–GCs than those of N–GCs.

PFOA also shows consistent binding affinity for progesterone [242] dysregulating progesterone-activating genes in endometrial cells. This study was conducted in cohorts of 146 exposed females aged 18–21 from the Veneto region in Italy and 1080 non-exposed cohorts. The dysregulation of the genetic cascade leading to embryo implantation and endometrial receptivity was significant, a sad finding since the endometrium clearly represents an important fertility determining factor. Melatonin plays an antioxidant role in protecting luteinizing granulosa cells from ROS, therefore enhancing progesterone production in the follicle during ovulation [243]. Indeed, progesterone production can be inhibited by H_2_O_2_ which production can be induced by the presence of pollutants. In the end PFAS (PFOA in particular) could induce female infertility compromising progesterone production and enhancing ROS production and also interfering with progesterone hormonal activity.

### 5.3. Pregnant Women and Developmental Consequences

ROS have both physiologic and pathologic roles in placenta, embryo, and fetus. The development of the embryo occurs in a relatively low-oxygen environment and this structure has low antioxidant capacity. As placentation progresses, there is an increased oxygen exchange with maternal blood, with an increased the cellular generation of ROS.

PFAS are bioaccumulative pollutants, and prenatal exposure to PFAS is believed to impact human fetal development and may have long-term adverse health effects later in life. Mamsen et al. [244] had monitored the concentrations of 5 PFAS molecules (PFOA, PFOS, PFNA, PFUnDA, and PFDA) in human fetus, placentas, and maternal plasma to evaluate to what extent these compounds were transferred from mother to fetus. PFAS concentration in fetal organs increase with fetal age. In maternal plasma, PFOS was detected at the highest concentrations, followed by PFOA, PFNA, PFUnDA, and PFDA, whereas in placentas and fetal organs the concentrations were greatly reduced, compared to maternal plasma.

Sagiv et al. [245] measured plasma concentrations of 4 PFAS in early pregnancy among 1645 women in Project Viva, a study of a birth cohort recruited during 1999–2002 in Eastern Massachusetts. They concluded that concentrations of early-pregnancy PFOS, PFOA, and PFNA were inversely associated, albeit modestly, with fetal growth and gestation length in Project Viva.

However, all the evaluated PFAS were transferred from mother to fetuses indicating that fetuses were systematically exposed to these compounds, even if not with the same level of danger because of these molecules pass from the mother to the fetus with different efficiencies [246,247,248,249], while another study conducted in China did not found any significant association [250].

Infant and children health is related to maternal characteristics such as maternal age and lifestyle during or before pregnancy. People may be exposed to PFAS in their living environment, through contaminated food, food packaging, and drinking water [251,252,253,254,255,256,257,258], but still many results are inconsistent.

PFAS levels varied between different maternal characteristics including maternal delivery age, pre-pregnant BMI, parity, maternal education level, smoking status, alcohol consumption history, and annual household income. Numerous epidemiological studies have reported associations between prenatal PFAS exposure and adverse health outcomes [259].

Some of these compounds are suspected to impact fetal development, and may disturb the endocrine system [260,261,262,263] or cause or pregnancy loss [264].

Prenatal exposure to PFOA has been associated with a dose-dependent decrease in birth weight [265,266], reduction in birth length [267], reductions in abdominal circumference [267], ponderal index [265], and also reduction in head circumference [265].

Prenatal exposure to PFAS has been associated with immunomodulation and may contribute to the etiology of asthma, a common non-communicable disease in children. Recently, higher serum-PFAS concentrations in childhood have been associated with increased odds of concurrent asthma [268], perhaps due to a shift towards a T-helper type 2 cell (TH2) immune response [269] as seen in allergic asthma and allergic diseases. However, other studies could not replicate this association [224,270]. Furthermore, measles, mumps and rubella (MMR) vaccination in early life may have a protective effect against asthma or asthma symptoms [271], an association that was previously substantiated in other analyses [272]. The protective effect is possibly due to the elicitation of a TH1-biased response [273], and it is possible that a TH1-biased response induced by MMR vaccination could suppress a PFAS induced TH2 immune response thus minimizing the effect of PFAS on asthma among MMR-vaccinated children.

Mora et al. [274] suggest that prenatal and mid-childhood PFAS exposure may be associated with modest, but somewhat conflicting changes in the lipid profile and ALT levels in children, particularly among girls. They also found that higher prenatal and mid-childhood PFOS, PFOA, and/or PFDeA concentrations were associated with some beneficial changes in the lipid profile, including slightly higher HDL–C, lower triglycerides (TG), and/or lower total cholesterol (TC)/HDL–C ratio, again mainly among girls.

In rodents, exposure to high doses of PFOS and PFOA during pregnancy reduced postnatal survival, birth weight, growth of the pups, and increased disturbed lactation [275].

Notably, the half-lives of PFAS in rats are few days short [276] compared to the 200 days in the cynomolgus monkey [80], and the 2.5–4.5 years in humans [277], suggesting a large difference in the elimination kinetics between species, and animal models may, therefore, only reflect the human situation to a limited extent.

### 5.4. Kidney and Thyroid Disorders

The kidney is another target of PFAS as it is involved in its excretion and it has been hypothesized that PFAS may damage kidneys via reabsorption of PFAS across the renal tubules. This reabsorption is hypothesized to occur due to renal tubule efflux transporters that actively transport PFAS back into systemic circulation, contributing to their long half lives in the human body [278,279].

Although the mechanisms of toxicity are not well understood for each individual PFAS, it is possible that individual compounds impact target tissues via different modes of action. For example, peroxisome proliferator alpha (PPARα) is a suspected nuclear receptor target of PFAS and is expressed in the liver and kidney, however the extent to which PFAS activate PPARα is thought to vary by carbon chain length and functional group, with some PFAS exhibiting high levels of PPARα activation (e.g., PFOA) and others exhibiting none (e.g., PFDeA) [280]. It is possible that some PFAS alter kidney function via activation of nuclear receptor PPARα and others may exert their effects through other toxicological mechanisms such as mitochondrial dysfunction [281]. As already said, PPARα can be involved in the conversion of ROS in less harmful compounds (reduction of hydrogen peroxide) which potentially can be produce by the presence of xenobiotics like PFASs.

Toxicology studies by Stanifer et al. [282] demonstrated associations between PFAS exposure and lower kidney function and/or kidney cancer. Pharmacokinetic studies demonstrated that kidneys were major routes of elimination, with active proximal tubule transport. In several studies, PFAS exposure altered several pathways linked to kidney diseases, including oxidative stress pathways, peroxisome proliferators-activated receptor pathways, nuclear factor erythroid 2-related factor (Nrf2) pathways, partial epithelial mesenchymal transition, and enhanced endothelial permeability through actin filament modeling.

It is possible that the relationship between the thyroid hormone and glomerular filtration rate (GFR) may contribute to the associations with serum PFAS. Reduced GFR is a consequence of hypothyroidism whereas increased GFR is a consequence of hyperthyroidism [283]. It is possible that PFAS indirectly affect GFR by disrupting the thyroid, or that PFAS affect the thyroid and kidney independently, or that reverse causation stemming from thyroid disease-related alterations in GFR accounts for the associations reported by Blake et al. [278]. A combination of reverse causality with respect to GFR and true adverse effect on the thyroid could be responsible for the associations reported by the same authors

Verner et al. [284] have evaluated how much of the epidemiologic association between prenatal exposure to PFAS and reduced birth weight might be attributable to confounding by GFR. Their results suggest that GFR drives a portion of this association, but not all of it, and that its influence becomes more important with increasing gestational weeks.

### 5.5. Cholesterol

PFAS exposure may affect metabolic functions. The possible binding of PFAS to PPARs and other nuclear receptors raises concerns that PFAS may affect cholesterol levels [285]. Previous studies have reported that PFAS may elevate cholesterol levels in animals, as illustrated by the decreased TC in serum occurred in the 0.75 mg·kg^−1^/day dose group at serum PFOS levels > 100 ppm in Cynomolgus Monkeys [80]. However, when using the animal data, the relevance between human and animal response to adverse health effects should be clarified. Given the apparent between-species differences in pharmacokinetics and tissue distribution of PFAS, as well as functional and structural differences in PPARs [16,80,81,286], caution must be taken when extrapolating data regarding PFAS from animal studies to humans. Steenland et al. [287] suggested that higher serum PFOS was associated with higher levels of TC, LDL, and TG. Contrastingly, in a cross–sectional study conducted by Olsen and Zobel [288], PFOA was not significantly associated with TC or LDL. Thus, the association between cholesterol levels and PFAS exposure are not consistent among the human studies [289].

Positive correlations among PFOA, PFOS, and total cholesterol were addressed in both Dong et al. and Nelson et al.’s studies [290,291].

A statistically significant and positive association between self-reported high cholesterol and PFNA exposure was observed, consistent with reports that other PFAS may affect blood lipid levels [292].

Jain and Ducatman [293] hypothesized that the combination of PFAS exposure and obesity increases the risk of alteration of lipid metabolism. Lipids are the class of biological molecules most susceptible to be attacked by ROS. Oxidation occurs on the fatty acids present in cell membranes or lipoproteins and, as the number of double bonds present in the molecule increases, their susceptibility to oxidation increases as well. Peroxidation leads to the formation of secondary products such as aldehydes and ketones, recognized as toxic or carcinogenic. Following the oxidation of the thiol groups of some amino acids and the release of Fe by degradation of porphyrin rings, the proteins lose their physiological structure and consequently their functionality.

### 5.6. Diabetes

PFAS are structural homologs of fatty acids, and scientific evidence suggests that PFAS can disrupt metabolism and endocrine function [294,295]. Accumulating evidence has also suggested that PFAS may also interfere with human metabolism through PPAR independent pathways, as mentioned in the previous paragraph. As example of this is how PFOA alters expression of proteins in human liver cells that are regulated by hepatocyte nuclear factor 4a [296], which is a key regulator of lipid metabolism and gluconeogenesis [297,298] as well as being involved in thyroid hormone homeostasis [299]. In particular, emerging evidence suggests that PFAS are endocrine disruptors and may contribute to the etiology of type 2 diabetes (T2D).

Several epidemiological studies have investigated the association between exposure to PFAS, diabetes incidence, and markers of both metabolism and glycemia with inconsistent results.

Sun et al. [300] have examined the associations between PFAS exposures and subsequent incidence of T2D in the Nurses’ Health Study II (NHSII) and evaluated potential demographic and lifestyle determinants of plasma PFAS concentrations. After multivariate adjustment for T2D risk factors, including body mass index, family history, physical activity, and other covariates, higher plasma concentrations of PFOS and PFOA were associated with an elevated risk of T2D. Strong correlations between PFAS levels and established diabetes risk markers, such as adiponectin, insulin, or HbA1c, were not observed, although the cross-sectional nature of these correlations has excluded causal inference. These findings support a potential diabetogenic effect of PFAS exposures, especially PFOS and PFOA. Oxidative stress and estrogenic effects observed in experimental studies are among other possible modes of action that may explain the PFAS–T2D associations. In addition, it is possible that PFAS have stronger effects among individuals at a higher risk of diabetes (e.g., overweight) or during periods of weight change (e.g., growth spurts in childhood and puberty) [272,301].

Associations for markers of insulin resistance and metabolism are also conflicting across epidemiological studies. For example, in a cross-sectional analysis of a U.S. representative sample from NHANES 1999–2000 and 2003–2004, higher serum PFNA was associated with hyperglycemia but with lower risk of metabolic syndrome, while PFOA was associated with higher β-cell function, and PFOS was associated with higher insulin, HOMA–IR, and β-cell function [220]. However, an analysis limited to the 2003–2004 NHANES cycle found no evidence of an association of PFAS with HOMA–IR [291]. Similarly, in the 2007–2008 Canadian Health Measures Survey, PFOS, PFOA, and PFHxS serum concentrations were not associated with plasma insulin, HOMA–IR, or metabolic syndrome [219]. In a study on Taiwanese adults, only PFOS was associated with higher glucose levels post-OGTT, but PFOA, PFNA, and PFUA were inversely associated with post glucose-load measurements, suggesting a protective effect [302]. In the elderly Swedish cohort, PFOA was positively associated with the ratio of proinsulin to insulin [303].

Another study has examined adults at high risk of T2D with plasma concentrations of several PFAS utilizing higher markers of insulin resistance and β-cell function at baseline: there was no strong evidence that baseline plasma PFAS influenced trajectories of insulin resistance and β-cell function during up to 4.6 y of follow-up [304].

Further studies are needed to elucidate whether effects of PFAS exposure may be age and/or sex dependent. Potential mechanisms underlying associations between PFAS and T2D risk are unclear.

### 5.7. Platelets and Cardiovascular Desease

The effect of ROS on platelet function in coronary heart diseases is complex and poorly defined. Oxidative stress is recognized as an important mediator of atherothrombotic events in cardiovascular diseases. It is known that ROS directly participate in the regulation of platelet activation and thrombus formation. Platelets themselves also generate ROS through several intracellular sources. However, the direct effects of ROS on platelets are reportedly varied.

De Toni et al. [305] investigated the effect of PFOA exposure on platelet function, a key player in the atherosclerosis process and in the development of acute thrombotic events. Their importance in coronary diseases and in acute coronary syndromes is indirectly confirmed by the benefit of antiplatelet agents in these disorders. Platelet reactivity is altered by a number of environmental factors, such as age, serum cholesterol, diabetes, catecholamine levels, cigarette smoking, obesity, and alcohol consumption. In addition, a role for platelets in the evolutionary phase of the atherosclerotic plaque has been suggested by the observation that platelets can promote foam cell formation even in the absence of hyperlipidemia.

In this study, PFOA accumulation in platelets, changes in platelet membrane fluidity and activation after dose-dependent exposure to PFOA were evaluated, intracellular calcium trafficking and platelets aggregation state was evaluated in 48 men living in a specific area of the Veneto region with high PFAS environmental pollution and compared with 30 low-exposure control subjects.

Platelet membrane was the major target of PFOA, whose dose-dependent accumulation was associated in turn with increased membrane fluidity. Most platelet agonists, including thrombin, ADP, and epinephrine, stimulate cell surface receptors that span the platelet membrane, representing a potential target for chemicals that induce alterations in membrane fluidity. These data let to hypothesize a major production of ROS after the interaction of PFAS with fatty acids metabolism and steroidogenic machinery, in particular interfering with NO production [187].

Exposed subjects had higher serum and platelet levels of PFOA, together with increased aggregation parameters, compared with controls. In the presence of PFOA, platelets increase their cytosolic calcium concentration. These data help to explain the emerging association between PFAS exposure and cardiovascular diseases.

## 6. PFAS and Oxidative Stress in Other Vertebrates

We wanted to dedicate this section to the PFAS-induced oxidative stress in non–human vertebrates. This choice is only apparently peculiar for a review that wants to focus on aspects related to humans, as many current scientific studies on non-human vertebrates are collecting data that may have important practical implications on human health. Certainly, most of them are using model organisms whose validity is universally recognized, among all the zebrafish (*Danio rerio*). But even those who study non-model animals are important, because those species constitute food products or because they are bioindicators of the environmental quality.

What emerges clearly from the literature data is that PFAS induce oxidative stress in animals. This claim is supported by chemical, biochemical, and molecular data (Table 2).

### 6.1. Chemical Data

The chemical data concern the formation of ROS and lipid peroxidation. In vitro studies, using goldfish (*Carassius auratus*) lymphocytes exposed to PFOA and freshwater tilapia (*Oreochromis niloticus*) hepatocytes treated with PFOS or PFOA, reveal that PFAS significantly increased ROS formation rate in a dose dependent manner [184,306].

In zebrafish experimentally exposed to various doses of PFOS, ROS formation increased significantly in embryos [307,308] and in liver of adult specimens, while in gills, intestines, and brain no significant changes were highlighted [309]. The rate of ROS formation also increased in zebrafish embryos and larvae exposed to PFNA [310], and to two novel PFOS alternatives, 6:2 fluorotelomer sulfonamide alkylbetaine (6:2 FTAB) [311] and 6:2 chlorinated polyfluorinated ether sulfonate (commercial name F–53B) [312].

Lipid peroxidation is reported in fathead minnow (*Pimephales promelas*) embryos and zebrafish larvae in response to PFOS [307,313], in goldfish lymphocytes and freshwater tilapia hepatocytes exposed to PFOA [184,306], and in zebrafish embryos under PFOS, PFOA, and PFNA [308,314]. An interesting result was obtained in fathead minnow, where PFOA was discovered having a different effect in males rather than females, the former showing a significant increase in damaged lipids but no such change was observed in female fish [315]. These data well correlate with the increase of ROS formation rate and suggest that antioxidant defense systems are not totally able to avoid oxidative damage to macromolecules and cell structures [316].

Exposure to PFOS alternatives gave mixed results. In fact, a decrease in lipid damage was highlighted in liver and larvae of zebrafish after F–53B exposure [317,318] but there was an increase in zebrafish embryos exposed to 6:2 FTAB [311].

The data of one of few papers that does not deal with fish, in which embryonic exposure of chicken (*Gallus gallus domesticus*) to PFOS and F–53B at environmentally relevant concentrations, bucked this trend. The treatment did not cause oxidative damage to lipids or proteins [319].

### 6.2. Biochemical Data

Biochemical data concern the biosynthesis of GSH and the induction of antioxidant enzymes, usually expressed as cellular and tissue enzymatic activity. Very few studies analyzed GSH levels in organisms exposed to PFAS, and they exclusively performed in vitro experiments. Among those, we mention one on goldfish lymphocytes treated with PFOA [306] and one on tilapia hepatocytes exposed to PFOS or PFOA [184]. In both cases, a decrease was observed in GSH content. The authors correlated these results to the detoxifying activity of enzymes such as GPx and GST, which use this tripeptide as co-factor. However, the situation is much more complex, because we must take into account not only the oxidation of GSH in GSSG but also its biosynthesis, catalyzed by γ–glutamyl–cysteine ligase (GCL) and glutathione synthetase, and GSSG depletion by Multidrug Resistance Protein 1 [320].

On the other hand, studies using zebrafish as a model organism, aimed to evaluate the toxicity of F–53B, are slightly more abundant. In larvae, the exposure to F–53B resulted again in a dose dependent decrease of GSH content [312,318]. Instead, the data relating to adult organisms show a differential response between sexes, with females presenting no significant difference in GSH content in liver of treated specimens compared to the controls [317].

Regarding the activity of antioxidant enzymes, the most analyzed proteins are SOD, CAT, and GPx. Exposure to PFAS leads to a generalized increase in SOD induction, which is reflected in an increased enzymatic activity. This occurs both in zebrafish treated with PFOS [307,308,309] and in non-model species used in laboratory treatments such as freshwater tilapia exposed to PFOA [184], or living in a PFAS polluted environment such as yellow perch (*Perca flavescens*) sampled from St. Lawrence River [321]. However, there are some exceptions, such as those highlighted with Japanese medaka (*Oryzias latipes*) and goldfish, in which exposure to PFOA did not change or decreased the SOD activity, respectively [306,322].

A non-unique effect is also produced by alternatives to PFOS. F–53B significantly decreased the SOD activity in zebrafish larvae and adults [312,317,318], and 6:2 FTAB did not produce variations in treated zebrafish embryos [311].

All these data refer to the activity of Cu, ZnSOD, while for mitochondrial SOD (SOD2) the only available data have been found in chicken, where the activity of this enzyme increased after the treatments with PFOS or F–53B [319].

SOD induction is probably related to an increase in the formation rate of superoxide radicals, that are the specific target of this enzyme, whereas a decrease in cellular or tissue activity (for SOD as well as other antioxidant enzymes) is indicative of a more or less strong oxidative stress condition, in which an inactivation of the antioxidant defenses begins to occur [323].

CAT activity showed even greater variability than SOD one, with responses that are not only species-specific but also dose dependent. For example, in environmental conditions, where relatively low concentrations of PFAS are usually found, there was an increase in CAT activity, as in the case of yellow perch [321]. Exposure to higher concentrations under laboratory conditions may lead to an increase of CAT activity, as in zebrafish embryos exposed to 0.2, 0.4, and 1.0 mg L^−1^ PFOS [307], but a further slight increase (1.6 mg L^−1^) can be sufficient to exert inhibitory effects characteristic of oxidative stress [308]. Other fish species, such as freshwater tilapia, seems to be more resistant to oxidative stress, with CAT activities that continue to increase even in specimens exposed to 30 mg L^−1^ of PFOS or PFOA [184]. However, very high PFAS concentrations lead to a sharp decrease in enzymatic activity, as observed in zebrafish and Japanese medaka specimens exposed to PFOA [281,322].

The route of acquisition of these pollutants could also influence the variations in CAT activity, and in particular the food route does not seem to be useful to favor oxidative stress, as highlighted in rainbow trout (*Oncorhynchus mykiss*) fed with a PFOA enriched diet [324].

Even in the case of CAT, there are different effects between F–53B and 6:2 FTAB. The first significantly decreases the CAT activity in zebrafish larvae and adults [312,317,318], the second produces incremental variations in treated zebrafish embryos [311]. In chicken, CAT activity was not significantly affected by the treatments with F–53B [319].

A similar variability was also observed for GPx. In some cases, exposure to PFAS did not produce any change in enzymatic activity, as in Japanese medaka after treatment with PFOA [322], in Arctic kittiwakes (*Rissa* sp.) that accumulate PFAS from the environment [325], or in chicken experimentally exposed to PFOS [319].

In other cases, an increase in GPx activity was observed, such as in zebrafish exposed to PFOS [307,308,309]. Instead, in freshwater tilapia was observed a dose dependent reduction of GPx activity after both PFOS and PFOA exposure [184].

Again, alternatives to PFOS produce variable effects, with F–53B that significantly induce GPx in zebrafish larvae and adults [312,317,318], and 6:2 FTAB that instead decreases the GPx activity in treated zebrafish embryos [311].

It is interesting to note that often the enzymatic activities of CAT and GPx are inversely correlated. This has a physiological significance, as both enzymes target H_2_O_2_, and therefore they can play a complementary role within the antioxidant system, ensuring adequate protection of the cell against the negative effects of inorganic and organic peroxides [326].

In the literature there are no data on the activity of Prdxs, which are enzymes that have only recently received great attention from the scientific community and are therefore still poorly studied. Instead, there are some data, even if very limited, on the GR. In freshwater tilapia, GR activity significantly increased after exposure to PFOS or PFOA [184]. In chicken, GR activity were not significantly affected by the treatments with PFOS or F–53B [319], as well as GCL. This enzyme is not directly part of the antioxidant system but plays an essential role in the detoxification of ROS as it catalyzes the biosynthesis of GSH [327].

### 6.3. Biomolecular Data

The biomolecular data concern the expression of mRNA, analyzed by microarray or quantitative Real Time PCR. Generally, a transcription up-regulation of various genes encoding antioxidant enzymes was observed. In Juvenile Atlantic salmon (*Salmo salar*) *sod*, *cat*, and *gpx* genes were significantly up-regulated in kidney and liver upon exposure to PFOA or PFOS [328]. In rare minnow (*Gobiocypris rarus*) an up-regulation of *gpx1*, *prdx,* and *msr* in specimens exposed to PFOA was reported [329,330]. In zebrafish *gpx1* and *prdx1* gene transcriptions were significantly up-regulated in larvae and embryos under PFAS treatment [314,331]. In yellow perch an over-transcription of *cat* gene was observed in specimens from a PFAS polluted environment [321]. In largemouth bass (*Micropterus salmoides*) living in a PFOS polluted environment was observed an activation of *gpx1a* in testis and *gpx4a* in liver [332]. PFOS also triggered a mild increase in *sod2* expression in chicken [319].

The activation of all these genes is probably related to a gene regulation that involves some enhancer sequences (antioxidant response elements, ARE) that are present in the gene promoters. This hypothesis is supported by the concomitant increase in mRNA expression of the gene that encodes the Nrf2 protein, the transcription factor that interacts with the ARE sequences, initiating gene transcription. The Nrf2 gene expression was significantly upregulated in liver of dark-spotted frog (*Pelophylaxnigro maculatus*) in response to PFOA [310], in zebrafish embryos upon exposure to PFOS [307], but also in larvae of this species exposed to F–53B [312].

However, the literature also reports other cases in which exposure to PFAS did not lead to an increase in the mRNA expression of the genes encoding antioxidant enzymes. For example, PFAS or F–53B exposure did not significantly affect the transcript levels of *sod1* in zebrafish larvae [312,331]. This is evidently an indication that under those experimental conditions there was no consistent implementation of ROS formation rate. A similar situation, but involving other antioxidant enzyme genes (*cat*, *gpx4*, *gr,* and *gcl*), was found in chicken, where the exposure to a low dose of PFOS or a high dose of F–53B did not significantly affect the mRNA expression [319].

Finally, there are still other cases in which there was even a decrease in gene expression. This occurred in zebrafish, where exposure to PFAS significantly altered the gene transcription of *sod1*, *cat*, *gpx1a,* and *prdx6* [281,308], in rare minnow, where *gpx* expression diminished in liver under treatment with PFOA [330], and in largemouth bass living in a PFOS polluted environment, where an inhibition of *sod2*, *cat*, *prdx1*, *prdx3,* and *prdx4* was observed in various organs [332]. These inhibitions may be the result of a negative feedback control of gene expression but also of a toxic effect that can affect the activity of Nrf2.

These data, in addition to hint out a complex regulatory system that differentially involves the various antioxidant enzymes, highlight a feature of gene expression regulation already found for other antistress proteins. In fact, in many cases there was no correlation between the variation of active protein biosynthesis and the increase of mRNA expression. This may certainly be due to the different half-lives of these two types of molecules, but it is also known that mRNAs of stress proteins can be stored in cytoplasmic foci, such as P bodies or stress granules (SG), where they undergo degradation or future translation, respectively [333,334]. This condition is a common trait in organisms adapted to have a rapid response to the presence of a new stressor [335,336,337].

**Table 2:** PFAS effects on human and other vertebrates.

Species	PFAS	Effects	References
*Carassius auratus*	PFOA	Increased ROS formation ^1^, lipid peroxidation ^2^, GSH decrease ^3^, SOD induction ^4^, CAT induction ^4^, GPX inhibition ^4^, GR induction ^4^	[184]
*Danio rerio*	PFOA	CAT inhibition ^7^, *cat* down-regulation ^8^, *prdx* down-regulation ^8^	[281]
*Danio rerio*	PFOS	Increased ROS formation ^1^, lipid peroxidation ^2^, SOD induction ^4^, CAT induction ^4^, GPX induction ^4^, *nrf2* up-regulation ^5^	[307]
*Danio rerio*	PFOS	Increased ROS formation ^1^, lipid peroxidation ^2^, SOD induction ^4^, CAT inhibition ^4^, GPX induction ^4^, *sod* down-regulation ^5^, *cat* down-regulation ^5^, *gpx* down-regulation ^5^	[308]
*Danio rerio*	PFOS	Increased ROS formation ^1^, SOD induction ^4^, GPX induction ^4^	[309]
*Danio rerio*	PFNA	Increased ROS formation ^1^	[310]
*Danio rerio*	PFOA, PFNA	Lipid peroxidation ^6^, *gpx* up-regulation ^4^, *prdx* up-regulation ^5^	[314]
*Danio rerio*	PFPiAs	*gpx* up-regulation ^5^, *prdx* up-regulation ^5^, *msr* up-regulation ^5^	[331]
*Gallus gallus domesticus*	PFOS	SOD induction ^4^	[319]
*Gobiocypris rarus*	PFOA	*gpx* down-regulation ^5^, *prdx* up-regulation ^5^, *msr* up-regulation ^5^	[329]
*Gobiocypris rarus*	PFOA	*gpx* up-regulation ^5^	[330]
*Homo sapiens*	PFOA, PFOS	Increased ROS formation ^1^, apoptosis ^11^, SOD induction ^4^, CAT induction ^4^, GR induction ^4^, GPX inhibition ^4^	[192]
*Homo sapiens*	PFHxS, PFOA, PFOS, PFNA, PFDA, PFUnA, PFDoA	Increased ROS formation ^1^, increased DNA damage ^9^, antioxidant defense inhibition ^10^	[196]
*Homo sapiens*	PFOA, PFOS	Estrogenicity and anti-estrogenicity ^12^	[202]
*Homo sapiens*	PFOA, PFOS, PFNA	Estradiol production ^13^, progesterone ^13^ production, testosterone production ^13^	[203]
*Homo sapiens*	PFOA, PFOS	Thyroid diseases	[213]
*Homo sapiens*	PFOA, PFOS	Chronic kidney diseases	[216]
*Homo sapiens*	PFHxS, PFOA, PFOS, PFNA	Decreased eGFR ^14^	[217]
*Homo sapiens*	PFHxs, PFOA, PFOS	Increased cholesterol outcomes ^15^	[218]
*Homo sapiens*	PFOA, PFOS, PFNA	Hyperglycemia ^16^, increased serum HDL cholesterol ^16^, increased blood insulin ^17^	[219]
*Homo sapiens*	PFOA, PFOS	Increased blood levels of glucocorticoid ^17^, increased blood levels of androgenic hormones ^18^	[222]
*Homo sapiens*	PFOA, PFOS	Increased total adiponectin levels ^19^, body weight decrease	[223]
*Homo sapiens*	PFHxS, PFHxA, PFHpA, PFOA, PFOS, PFNA, PFDA, PFUnDA, PFDoDA, PFTrDA, PFTeDA	Allergic diseases	[224]
*Homo sapiens*	PFOA, PFOS	Polyunsuturated fatty acids levels decrease ^20^, birth weight decrease	[225]
*Homo sapiens*	PFOA	Up-regulation of progesterone activated genes ^5^	[242]
*Homo sapiens*	PFOA, PFOS	Increased T4 levels ^21^	[260]
*Homo sapiens*	PFOA	Reduced fetal growth	[261]
*Homo sapiens*	PFOA, PFOS	Reduced birth weight	[262]
*Homo sapiens*	PFOA, PFOS	Reduced cord serum concentrations and birth weight size	[265]
*Homo sapiens*	PFOA, PFOS	Affected growth of organs and the skeleton	[267]
*Homo sapiens*	PFBS, PFHxA, PFHpA, PFHxS, PFOA, PFOS, PFNA, PFDA, PFTA, PFDoA	Asthma	[268]
*Homo sapiens*	PFHxS, PFOA, PFOS, PFNA	Asthma	[270]
*Homo sapiens*	PFOA, PFOS	High insulin and trygliceride concentrations	[272]
*Homo sapiens*	PFOA, PFOS, PFDeA	Changes in lipid profile ^16^, changes in ALT levels ^16^	[274]
*Homo sapiens*	PFOA, PFOS, PFNA, PFHxS, PFDeA, PFOSA, Et-PFOSA, Me-PFOSA	Increased TSH^22^, increased eGFR ^14^	[278]
*Homo sapiens*	PFOA, PFOS	Reduced birth weight	[284]
*Homo sapiens*	PFOA, PFOS	Increased cholesterol outcomes ^16^	[287]
*Homo sapiens*	PFOA, PFOS, PFNA, PFHxS	High serum cholesterol ^16^	[290]
*Homo sapiens*	PFOA, PFOS, PFNA, PFHxS	High serum cholesterol ^16^	[291]
*Homo sapiens*	PFOA	HNF4α inhibition ^23^, *hnf4α* down-regulation ^5^	[296]
*Homo sapiens*	PFOS, PFOA, PFHxS, PFNA, PFDA	Potential diabetogenic effect	[300]
*Homo sapiens*	PFOA, PFOS	Potential adiposity, decreased beta-cell function	[301]
*Homo sapiens*	PFOA	Impaired platelet aggregation and increased cardiovascular risk	[305]
*Homo sapiens, Mus musculus*	PFPA, PFHpA, PFOA, PFUnA, PFDoA	Increased PPARα activity ^24^	[65]
*Homo sapiens, Mus musculus*	PFOA, PFOS, PFHxS, PFHxA, PFNA, PFDA, PFBA, PFBS	Increased PPARα activity ^24^	[280]
*Homo sapiens, Mus musculus, Rattus norvegicus*	PFOA, PFOS	Increased PPARα activity ^24^, increased PPARβ activity ^24^, increased PPARγ activity ^24^	[211]
*Homo sapiens, Mus musculus, Rattus norvegicus*	PFOA, PFOS	Reduced birth weight	[266]
*Homo sapiens, Rattus norvegicus*	PFOA, PFOS	Up-regulation of multiple nuclear receptor genes ^5^	[285]
*Micropterus salmoides*	PFOS	*gpx* up-regulation ^8^, *sod* down-regulation ^8^, *cat* up-regulation ^8^, *prdx* down-regulation ^8^	[332]
*Mus musculus*	PFOA	Lipid peroxidation ^25^, amylase induction, lipase induction, *sod1* up-regulation ^5^, *sod2* up-regulation5, *gpx2* up-regulation ^5^, *nqo1* up-regulation ^5^	[204]
*Mus musculus*	PFOS	Increased in peroxisomal fatty acid beta-oxidation ^4^, increased peroxisomal catalase activity ^4^	[208]
*Mus musculus*	PFOA	pparα up-regulation ^5^, pparγ up-regulation ^5^	[209]
*Mus musculus*	PFOS	Serum testosterone decrease ^19^, epididymal sperm counts decrease, down-regulation of genes encoding testicular receptors for gonadotropin, growth hormone, insulin-like growth factor 1 and steroidogenic enzymes ^5^	[234]
*Mus musculus*	PFOA	Damaged seminiferous tubules, reduced sperm quality, reduced serum testosterone and progesterone levels ^13^, INSL3 enzyme decrease ^23^, cytochrome P450 decrease ^23^	[235]
*Mus musculus*	PFOS	Immune response induction ^19^	[269]
*Oncorhynchus mykiss*	PFOA	CAT inhibition^4^	[324]
*Oreochromis niloticus*	PFOA, PFOS	Increased ROS formation ^1^, lipid peroxidation ^2^, GSH decrease ^3^	[306]
*Oryzias latipes*	PFOA	SOD inhibition ^4^, CAT inhibition ^4^	[322]
*Pelophylaxnigro maculatus*	PFOA	*nrf2* up-regulation ^5^	[313]
*Perca flavescens*	PFHxS, PFOS, PFDS, PFECHS, PFOA, PFNA, PFDA, PFUnA, PFDoA, PFTriA, PFTetraA	SOD induction ^4^, CAT induction ^4^, *cat* down-regulation ^5^	[321]
*Pimephales promelas*	PFOA, PFOS	Lipid peroxidation ^6^	[315]
*Rattus norvegicus*	PFOA	Increased ROS formation ^1^, increased mithocondrial superoxide formation ^25^, increased nitric oxide formation ^1^, apoptosis ^19^, increased proinflammatory cytokines ^19^, reduced adenosine triphosphate levels ^26^, reduced cardiolipin peroxidation ^27^, reduced cytochrome c release ^19^	[200]
*Rattus norvegicus*	PFOA	Increased ACO activity ^4^, increased PPARα activity ^24^	[207]
*Rattus norvegicus*	PFDoA	Serum testosterone decrease ^19^, serum estradiol decrease ^19^, down-regulation of genes involved in cholesterol transport and steroid biosynthesis ^5^	[233]
*Rattus norvegicus*	PFOA	Up-regulation of genes involved in metabolism of lipids, cell communication, growth, hormone regulatory pathways, proteolysis, peptidolysis and signal transduction ^8^, down-regulation of genes involved in inflammation and immunity, regulation of hormones, general metabolism and G-protein coupled receptor protein signaling pathways ^8^	[294]
*Salmo salar*	PFOA, PFOS	*sod* up-regulation ^5^, *cat* up-regulation ^5^, *gpx* up-regulation^5^	[328]
*Ursus marittimus*	PFASs	Increased levels of brain steroid hormones ^28^	[295]

^1^ 2′,7′-dichlorofluorescein deacetate; ^2^ malondialdehyde; ^3^ 4-chloro-1-methyl-7-trifluoromethylquinolinium methylsulfate; ^4^ enzymatic activity; ^5^ quantitative real-time PCR; ^6^ thiobarbituric acid reactive species; ^7^ MALDI-TOF-TOF mass spectrometry; ^8^ microarray; ^9^ comet assay; ^10^ total antioxidant capacity; ^11^ FACSort flow cytometry; ^12^ E-SCREEN bioassay; ^13^ Coat-a-count radioimmunoassay; ^14^ serum creatinine; ^15^ Ortho VITROS Clinical Analyzer; ^16^ enzymatic assay; ^17^ immunoenzymometric assay; ^18^ liquid chromatography-tandem mass spectrometry; ^19^ Enzyme Linked ImmunoSorbent Assay; ^20^ gas chromatography-mass spectrometry; ^21^ AutoDELFIA fully automated system; ^22^ isometric assay; ^23^ Wester blotting; ^24^ transactivation assay; ^25^ MitoSOX™ Red mitochondrial superoxide indicator; ^26^ ATP colorimetric assay; ^27^ 10-N-nonyl-Acridine Orange; ^28^ mass spectrometry.

## 7. Conclusions

Although many PFAS are no longer used in industrial activities, these chemicals are expected to pose a serious threat to humans and the environment in which they live for many years to come, because of their bioaccumulative properties and environmental persistence. In parallel, the production and use of replacement chemicals, some of which may already have been under production for decades, has been on the rise [338]. Among these, the F-53B component 6:2 chlorinated polyfluoroalkyl ether sulfonate (6:2 Cl-PFESA) has attracted attention since 2013 when the first report on its persistence, toxicity, and environmental occurrence was published [339]. Hexafluoropropylene oxide dimer acid (HFPO-DA; Gen-X) and dodecafluoro-3H-4,8-dioxanonanoate (ADONA) have also been the subject of several monitoring surveys of surface water and drinking water [340]. However, limited data are available regarding the environmental fate and ecotoxicology of such alternatives [338,341].

The available literature on PFAS shows that this group of chemicals causes a wide range of adverse outcomes in humans, most of which are related to oxidative stress, but there are still significant gaps in the current knowledge on the toxicity of these compounds. In particular, the knowledge on mechanisms by which PFAS act in generating cell toxicity is still very limited, starting from the role that is played by PPARs in the activation of cellular signaling when they bind and are activated by PFAS [342]. Therefore, future studies should focus on these gaps, in order to better counteract the negative consequences of PFAS on people exposed to these pollutants, but also on ecosystems, whose health and biodiversity of species is not at all unrelated to survival of the human species.

## Figures and Tables

**Table 1:** A list of the most common perfluorinated substances (PFAS) with their applications and properties [31].

PFAS	Use	Structure	Molecular Weight	Confirmed Toxic Effects
PFBA	synthetic chemistry	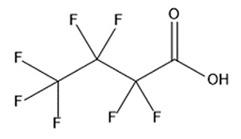	214.04	unknown
PFPeA	breakdown product of stain and grease-proof coatings on food packaging, couches, carpets	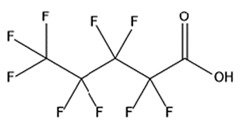	264.05	yes [61]
PFHxA	unknown	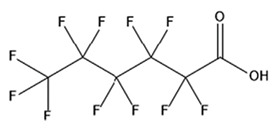	314.05	yes [62]
PFHpA	breakdown product of stain and grease-proof coatings on food packaging, couches, carpets	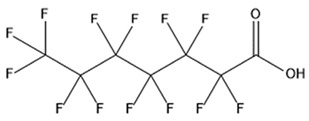	364.06	yes [63,64,65]
PFOA	water and oil repellant in fabrics and textiles, food packaging	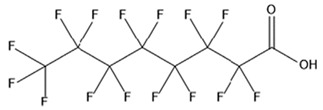	414.07	yes [66,67,68]
PFNA	surfactant for synthesis of textiles and polymers	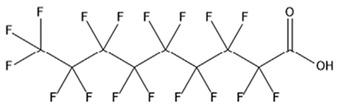	464.08	yes [61,69,70,71]
PFDA	breakdown product of stain and grease-proof coatings on food packaging, couches, carpets	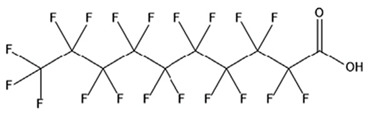	514.08	yes [72,73]
PFUnA	unknown	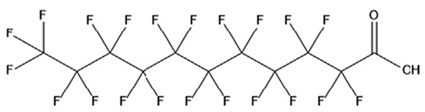	564.09	yes [74]
PFDoA	unknown	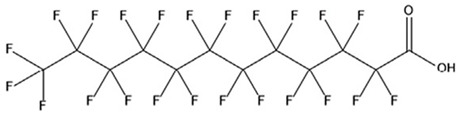	614.1	yes [75,76,77]
PFBS	stain repellant	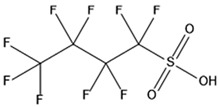	300.1	yes [78]
PFHxS	surfactant for textiles	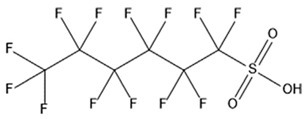	400.11	unknown
PFOS	firefighting foam, textiles	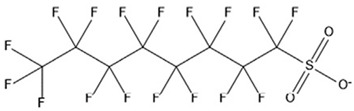	500.13	yes [79,80,81]

## Abbreviations

acyl CoA oxidase	ACO
Alkaline Phosphatase	ALP
Aminotransferase	ALT
Androgen Receptor	AR
Bisphenol A	BPA
Catalase	CAT
Cyclooxygenase 2	COX–2
Dichlorodiphenyldichloroethylene	DDE
Endocrine Disruptors	ED
Glutamyl-Cysteine Ligase	GCL
Glomerular Filtration Rate	GFR
γ–glutamyl transferase	GGT
Glutathione peroxidase	GPx
Glutathione Reductase	GR
Glutathione S-transferase	GST
Hepatocyte nuclear factor 4 alpha	HNF4α
Health Protection Agency	HPA
insulin like factor 3	INSL3
Luteinizing Hormone	LH
Methionine sulfoxide reductases	Msr
Normal Granulosa Cells	N–GCs
National Health and Nutrition Examination Survey	NHANES
Nurses’ Health Study II	NHSII
Nitric Oxide	NO
Polychlorinated Biphenyls	PCBs
Granulosa Cells from Polycystic Ovaries	PCO–GCs
Perfluorinated Alkyl Substances	PFAS
Perfluorinated Carboxylic Acids	PFCA
Perfluorinated compounds	PFCs
Perfluorobutanoic acid	PFBA
Perfluorobutane sulfonate	PFBS
Perfluorodecanoic acid	PFDA
Perfluorododecanoic acid	PFDoA
Perfluoroheptanoic acid	PFHpA
Perfluorohexanoic acid	PFHxA
Perfluorohexanesulfonic acid	PFHxS
Perfluorononanoic acid	PFNA
Perfluorooctanoic acid	PFOA
Perfluorooctane sulfonic acid	PFOS
Perfluoropentanoic acid	PFPeA
Perfluoroalkyl phosphinic acid	PFPiAs
Perfluoroundecanoic acid	PFUnA
Persistent Mobile Organic Contaminants	PMOC
Peroxisome Proliferator-Activated Receptor	PPAR
Peroxiredoxins	Prdx
Reactive Oxygen Species	ROS
Stress Granules	SG
Superoxide Dismutase	SOD
Type 2 Diabetes	T2D
Thyroxine	T4
Total Antioxidant Capacity	TAC
Total Cholesterol	TC
Tolerable Daily Intake	TDI
Triglycerides	TG
